# Complement C3b contributes to *Escherichia coli*-induced platelet aggregation in human whole blood

**DOI:** 10.3389/fimmu.2022.1020712

**Published:** 2022-12-14

**Authors:** Anne Landsem, Åse Emblem, Corinna Lau, Dorte Christiansen, Alexandra Gerogianni, Bård Ove Karlsen, Tom Eirik Mollnes, Per H. Nilsson, Ole-Lars Brekke

**Affiliations:** ^1^ Research Laboratory and Department of Laboratory Medicine, Nordland Hospital Trust, Bodø, Norway; ^2^ Linnaeus Centre for Biomaterials Chemistry, Linnaeus University, Kalmar, Sweden; ^3^ Department of Chemistry and Biomedicine, Linnaeus University, Kalmar, Sweden; ^4^ Centre of Molecular Inflammation Research, Norwegian University of Science and Technology, Trondheim, Norway; ^5^ Department of Immunology, Oslo University Hospital, University of Oslo, Oslo, Norway; ^6^ Department of Clinical Medicine, UiT The Arctic University of Norway, Tromsø, Norway

**Keywords:** platelet aggregation, glycoprotein IIb/IIIa, complement component C3, complement receptor 1, *Escherichia coli*, multiplate aggregometry, thromboinflammation

## Abstract

**Introduction:**

Platelets have essential functions as first responders in the immune response to pathogens. Activation and aggregation of platelets in bacterial infections can lead to life-threatening conditions such as arterial thromboembolism or sepsis-associated coagulopathy.

**Methods:**

In this study, we investigated the role of complement in *Escherichia coli* (*E. coli)*-induced platelet aggregation in human whole blood, using Multiplate^®^ aggregometry, flow cytometry, and confocal microscopy.

**Results and Discussion:**

We found that compstatin, which inhibits the cleavage of complement component C3 to its components C3a and C3b, reduced the *E. coli*-induced platelet aggregation by 42%-76% (p = 0.0417). This C3-dependent aggregation was not C3a-mediated as neither inhibition of C3a using a blocking antibody or a C3a receptor antagonist, nor the addition of purified C3a had any effects. In contrast, a C3b-blocking antibody significantly reduced the *E. coli*-induced platelet aggregation by 67% (p = 0.0133). We could not detect opsonized C3b on platelets, indicating that the effect of C3 was not dependent on C3b-fragment deposition on platelets. Indeed, inhibition of glycoprotein IIb/IIIa (GPIIb/IIIa) and complement receptor 1 (CR1) showed that these receptors were involved in platelet aggregation. Furthermore, aggregation was more pronounced in hirudin whole blood than in hirudin platelet-rich plasma, indicating that *E. coli*-induced platelet aggregation involved other blood cells. In conclusion, the *E. coli*-induced platelet aggregation in human whole blood is partly C3b-dependent, and GPIIb/IIIa and CR1 are also involved in this process.

## Introduction

The interaction between the immune and the hemostatic system in thromboinflammation is highly relevant in many diseases, and platelets tightly link these systems together (1). The role of platelets in hemostasis is well established, and during the last decades, the understanding of their contribution to both innate and adaptive immunity has expanded (2). Stroke, sepsis, and myocardial infarction are examples of diseases where platelets are central due to their important immune functions (3).

The interaction between platelets and bacteria is central in sepsis (4, 5). Bacteria and other pathogens can activate platelets (6) and induce platelet aggregation (7). Activated platelets can increase the defense against bacteria by limiting pathogen spreading, impede tissue invasion, secrete antimicrobial peptides, and trigger neutrophil extracellular traps. However, enhanced thrombus formation can be a significant threat to host organisms, as seen in sepsis, causing disseminated intravascular coagulation and organ failure (8, 9). The interaction between platelets and bacteria can be induced by various mechanisms; direct binding between bacterial surface proteins and receptors on platelets such as glycoprotein (GP) IIb/IIIa and GPIbα, and indirectly by binding of the plasma proteins fibrinogen and fibronectin that can attach to both bacteria and platelet receptors (10). In addition, bacterial toxins can interact with platelets (10). Platelets can bind, engulf and release antimicrobial peptides to kill the invaders (11).

The complement system is involved in bacteria-induced platelet aggregation (12, 13). Complement inhibition decreased *Cutibacterium acnes*-mediated platelet aggregation (12). *Escherichia coli (E. coli)*-induced platelet aggregation depended on plasma proteins; however, surface proteins on *E. coli* were most likely not involved in the aggregation (13). Notably, the different *E. coli* strains act differently concerning activation and interaction with platelets. Platelets activated with the non-pathogenic *E. coli* K12 displayed increased P-selectin and GPIIb/IIIa expression, in contrast to the pathogenic *E. coli* O18:K1, which did not increase these markers (14). Complement component C3a has been suggested to play a part in platelet aggregation (15, 16), but the detailed mechanisms regulating the interactions between complement, *E. coli*, and platelets are still unknown.

Platelets are closely connected to the complement system and express several complement receptors on the surface, including complement receptor (CR) 3 (17), CR4 (18), globular C1q receptor (gC1qR), collectin receptor (cC1qR), and the anaphylatoxin receptors complement component C3a receptor (C3aR) and complement component C5a receptor 1 (C5aR1) (19, 20). Platelets also express complement regulators such as CD46, CD55, and CD59 (21) and further recruits fluid phase regulators, like factor H and C4-binding protein, after activation (22). Platelet precursors, the megakaryocytes, produce C3, which differs from plasma C3 in electrophoretic mobility (23). The same C3 is found in platelet granules and is released by *E. coli-*induced activation (23). High level of plasma C3 is associated with an increased risk of venous thromboembolism (24). This is in accordance with a study showing that C3-deficient mice have a reduced incidence of venous thrombosis, reduced platelet deposition and reduced platelet activation *in vitro* (25). In contrast, C5-deficient mice showed no altered platelet activation *in vitro* or vessel wall platelet deposition *in vivo* (25). P-selectin, released from the platelet alpha granules, was shown to function as a C3b receptor, inducing complement activation on the surface of the platelets (26).

Platelets express about 50 000 GPIIb/IIIa receptor molecules on their surface, and fibrinogen can form a bridge between platelets by binding two GPIIb/IIIa molecules (27). The GPIIb/IIIa receptor has a non-binding conformation on the surface of resting platelets, and platelet activation triggers a conformational change of the GPIIb/IIIa binding site to a high-affinity state. Alpha and dense granules release increase the number of expressed GPIIb/IIIa receptors (28).

Complement inhibition is increasingly used as a therapeutic option in inflammatory, degenerative, and traumatic disorders (29–31), and it is important to understand how complement factors affect platelet aggregation. Although there is a growing understanding of the interaction between platelets and bacteria, the role of complement in this equation is largely unknown. In this study, we examined the interaction between platelets, complement, and *E. coli* bacteria in a human whole blood model (32).

## Methods

### Whole blood model

The whole blood model was performed as described previously (32). Healthy blood donors signed written informed consent. The study was approved by the regional ethics committee of the Northern Norway Regional Health Authority. Blood was anticoagulated with a highly specific thrombin inhibitor, lepirudin (Refludan^®^, Celgene, Uxbridge, UK) (final concentration 50 µg/mL). All equipment, tips and reagents were endotoxin-free. Dulbecco’s phosphate-buffered saline with CaCl_2_ and MgCl_2_ (PBS, Merck. Darmstadt, Germany) or inhibitors were preincubated at 37°C with blood in polypropylene tubes (Nunc, Roskilde, Denmark). After 5 minutes of preincubation, PBS or activators were added to the tubes and incubated for the indicated time at 37°C. The human whole blood model preceded flow cytometric analysis, ELISA and confocal microscopy. The *in-house* program made for Multiplate^®^ impedance aggregometry was also based on this model.

### Activators

Heat-inactivated *E. coli* (strain LE392, ATCC 33572, American Type Culture Collection, Manassas, VA) was grown and washed as previously described (32). Purified human anaphylatoxin C3a (Hycult Biotech, Uden, The Netherlands), native human C3b (Merck) and adenosine diphosphate (ADP) (ADPtest, Roche Diagnostics GmbH, Mannheim, Germany) were also used as activators.

### Inhibitors and controls

The following selective complement inhibitors at the C3 level were used; the C3 inhibitor compstatin (Cp40) (a generous gift from Prof. John D. Lambris), LEAF™ and Ultra-LEAF™ Purified anti-human C3a/C3a(desArg)/C3 antibody (clone K13/16 Biolegend, San Diego, CA), C3a receptor antagonist (C3aRA) (Trifluoroacetate salt SB290157, Cayman Chemical, Ann Arbor, MI), and anti-human C3b/iC3b antibody (clone 3E7, Hycult Biotech). This antibody binds to an epitope on C3b and iC3b, and inhibits the formation of alternative pathway C3 convertase, thus, preventing C3b opsonization of surfaces (33). The following inhibitors were also included in the experiments; anti-human C5 antibody eculizumab (Alexion Pharma GmbH, New Haven, CT), the C5a receptor antagonist PMX53 (34) (a generous gift from Prof. Trent Woodruff), anti-human CD14 antibody r18D11 (35), and anti-human/rat P-selectin/CD62P antibody (polyclonal sheep IgG, R&D Systems, Minneapolis, MN). The blocking anti-CR1 antibody (clone 3D9) (36) (a generous gift from Prof. Ronald P. Taylor). The GPIIb/IIIa inhibitor tirofiban hydrochloride monohydrate (Merck) was used both in Multiplate^®^ and flow cytometry analyses. The compstatin control peptide (R&D Systems), a LEAF ™ Purified mouse IgG1k (Biolegend), and polyclonal sheep IgG (R&D Systems) were used as controls. The recombinant IgG2/4 antibody NHDL was used as an isotype control for the anti-human CD14 antibody (37).

### Aggregometry

The Multiplate^®^ Analyzer (Roche, Mannheim, Germany) was used to measure platelet aggregation. Aggregation is detected by changes in the impedance between the electrodes in the test cuvettes. Blood samples were collected in hirudin tubes (Sarstedt, Nümbrecht, Germany). In some experiments, whole blood was replaced by platelet-rich plasma (PRP) or isolated platelets. To produce PRP, hirudin, anticoagulant citrate dextrose solution A (ACD-A) (Vacuette, Greiner Bio-One GmbH, Kremensmünster, Austria) or sodium citrate 3.2% (Greiner Bio-One) tubes were centrifuged at 150 x g for 15 minutes at room temperature, without applying a brake. Instead of applying the Multiplate^®^ commercial tests and test setup, an *in-house* program was used: 200 µL 0.9% NaCl-solution, inhibitors and 200 µL blood were preincubated in the test cuvettes for five minutes before activator (*E. coli*) or PBS was added. The concentration of heat-inactivated *E. coli* was 5.3 x 10^9^/mL, corresponding to 0.025 platelets per bacterium, assuming a platelet concentration of 4 x 10^8^/mL in whole blood. *E. coli* was titrated up to 10^10^ bacteria per mL to find the concentration that gave the highest level of platelet aggregation (**Figure S1**). The accepted difference between the wires in the test cell was set to 25% and the measurement time to 30 minutes. The platelet aggregation was detected as aggregation units (AU) and plotted against time, generating the aggregation measurement as the area under the curve (AUC).

### Platelet isolation

Human platelets were isolated as earlier described (38). Briefly, human whole blood was sampled in polypropylene tubes with ACD-A and incubated at 37°C for 10 minutes before centrifugation at 160 x g for 15 minutes at 37°C without brake. The PRP was pipetted into a new tube and incubated for 10 minutes. The PRP was centrifugated at 1100 x g for 10 minutes at 37°C with the brake setting on. The platelets were washed using Tyrode’s buffer (pH 7.35) (38) and prostaglandin E1 (Merck, 2.8 mM) was added to avoid platelet activation before the analysis on the Multiplate^®^. The platelet concentration was measured by a CELL-DYN Sapphire hematology analyzer and the concentration was adjusted to 2 x 10^8^/mL with Tyrode’s buffer.

### Flow cytometry analysis

All flow cytometry analyses were executed after the human whole blood model. C3b opsonization of platelets and *E. coli* was measured using a NovoCyte flow cytometer (ACEA Biosciences, San Diego, CA). Human whole blood was activated by Alexa Fluor 633 (Thermo Fisher Scientific, Waltham, MA) stained *E. coli* (staining as described in (39)). The samples were incubated for zero or 10 minutes before the reaction was stopped by adding ethylenediamine tetraacetic acid (EDTA, 10 mM final concentration). The samples were incubated with staining buffer (PBS with 0.1% bovine serum albumin (BSA)) containing a BV605-labeled mouse anti-human CD61-antibody (clone VI-PL2, Becton Dickinson Bioscience, San Jose, CA) to identify platelets, a V450-labeled mouse anti-human CD45-antibody (clone H130, Becton Dickinson) to identify leukocytes, and a FITC-labeled polyclonal rabbit anti-human C3c-antibody (Dako, Santa Clara, CA), for 10 minutes in the dark. Then, samples were lysed and fixed by adding 500 µL BD Phosflow Fix/Lyse (Becton Dickinson) per 25 µL of blood for 10 minutes at 37°C. The samples were centrifuged at 600 x g for 6 minutes, and washed with staining buffer. The population of platelets was gated in a CD45/CD61 plot to exclude the leukocytes. Further gating was done in an Alexa 633/CD61 plot to separate platelets conjugated to *E. coli* from platelets not bound to bacteria. As a measure of C3b opsonization, these populations were analyzed for mean fluorescence intensity (MFI) of the FITC anti-C3c antibody. The antibody reacts with C3c and the C3c part of C3 and C3b.

The activation of the GPIIb/IIIa receptor on platelets was measured using a conformational neoepitope-specific, FITC-labeled mouse anti-human PAC-1 antibody (clone PAC-1, Thermo Fisher Scientific). In the human whole blood model, blood samples were incubated for 5 minutes with inhibitors and PBS or *E. coli* (1 x 10^9^/mL) before staining with the anti-human PAC-1 antibody and BV605-labeled mouse anti-human CD61-antibody (Becton Dickinson). The staining was performed at room temperature for 15 minutes in the dark. The samples were fixed with paraformaldehyde (Thermo Fisher Scientific) (0.5% final concentration) for 20 minutes before analysis. The platelets were gated in an SSC/CD61 plot, and the results were reported as MFI of FITC anti-human PAC-1 antibody for the selected platelet population.

*E. coli*-induced leukocyte-platelet conjugate formation was analyzed after the human whole blood model. The samples were incubated for 15 minutes with compstatin and PBS or *E. coli* (5 x 10^8^/mL) and fixed with PFA (0.25% final concentration, Thermo Fisher Scientific) for 4 minutes at 37°C. The staining was performed with Alexa Fluor 647 labeled mouse anti-human CD14-antibody (clone MϕP9, BD Pharmingen), V500 labeled mouse anti-human CD45-antibody (clone HI30, Becton Dickinson), FITC-labeled mouse anti-human CD61-antibody (clone RUU-PL 7F12, Becton Dickinson), BV650-labeled mouse anti-human CD15-antibody (clone HI98, Becton Dickinson) and PE-labeled mouse anti-human CD235a-antibody (clone JC159, Dako) in a staining buffer (Becton Dickinson) for 15 minutes at room temperature in the dark. Then, samples were lysed by adding 5 x Vol EasyLyse (Becton Dickinson) for 15 minutes at room temperature in the dark. The leukocyte populations were gated in an SSC/CD45 plot, and further gated in a CD14/CD15 plot to separate the granulocytes and monocytes. These populations were analyzed for MFI of the FITC anti-human CD61 antibody.

### Confocal microscopy

For confocal microscopy, we used the same protocol as for the C3b detection by flow cytometry, including the human whole blood model, except here, *E. coli* was unlabeled. A FITC-labeled polyclonal rabbit anti-mouse antibody (Dako) was used as an isotype control antibody. One drop of the sample was mounted on a Superfrost^®^ Plus Microscopy slide (Thermo Fisher Scientific) with ProLong^®^ Gold antifade reagent (Thermo Fisher Scientific). To generate the laser scanning confocal images, we used the Zeiss LSM-800 super-resolution microscope (Oberkochen, Germany) with a 63x oil immersion objective (Plan-apochromat 63x/1.40 oil DIC M27). The software used to capture the images was Zen blue version 2.6. For the super-resolution image, the imaging device was Airyscan and GaAsP-PMT detector with gain at 850 V (preset). Red BV was captured with excitation wavelength 280 nm/emission wavelength 618 nm detection wavelength 565-624 nm; Green FITC with excitation wavelength 495 nm/emission wavelength 519 nm, detection wavelength 400-558 nm. For the confocal images combined with DIC, the imaging device was GaAsP-PMT1 and ESID photodiode detector. Detector gain was 527 V and for the ESID detector gain was set to 3. Green FITC excitation wavelength 495 nm/emission wavelength 519 nm detection wavelength 450-543 nm DIC detection wavelength was 400 nm.

### Enzyme-linked immunosorbent assays ELISA

We performed Wielisa (Svar Life Science AB, Malmö, Sweden) according to the manufacturer’s protocols to determine the inhibitory effect of the C3b antibody on complement activation pathways. The platelet activation markers in plasma; P-selectin (Thermo Fisher Scientific) and β-thromboglobulin (βTG) (Elabscience Biotechnology Inc., Houston, TX) were also measured by ELISA, after experiments using the human whole blood model.

### Statistical analysis

GraphPad version 9.2.0 (GraphPad Software, San Diego, CA) was used for the statistical analyses. The normal distributed results are given as mean values, and the non-normally distributed results as median values. The normality of the results was checked using the Shapiro-Wilks test. A paired Student’s t-test was used to compare the un-activated and bacteria-or ADP-activated samples. One-way repeated measurements analysis of variance (ANOVA) with Dunnett’s multiple comparisons test was used to compare the results of samples with different inhibitors against the sample with PBS and activator. Non-normally distributed data were analyzed by Wilcoxon matched-pairs signed-rank test and the non-parametric Friedman test with Dunn’s multiple comparison test. A p-value < 0.05 was defined as statistically significant. The results from donors that did not respond to *E. coli-*induced activation were excluded because the study aimed to find a mechanism leading to *E. coli*-induced platelet aggregation.

## Results

### *E. coli*-induced platelet aggregation was reduced by C3 inhibition

**Figure 1:**
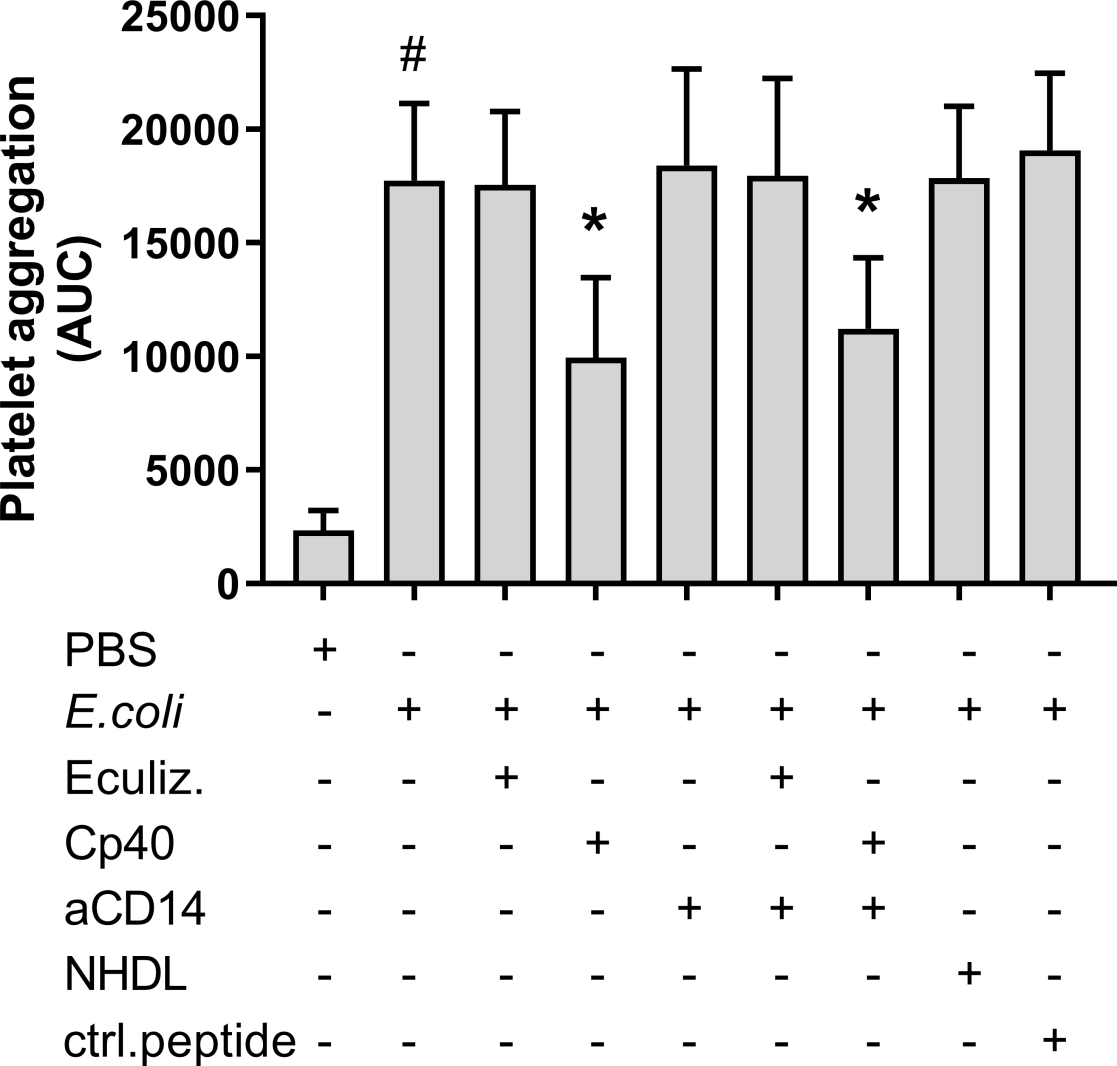
*Escherichia coli* (*E. coli*, 5.3 x 10^9^/mL*)-*induced platelet aggregation measured by Multiplate^®^ impedance aggregometry. To the samples were added phosphate-buffered saline (PBS) control, eculizumab (Eculiz, 100 µg/mL), compstatin (Cp40, 20 µM), anti-CD14 antibody (aCD14, 15 µg/mL), immunoglobulin G 2/4 control antibody (NHDL, 15 µg/mL) and control peptide (ctrl.peptide, 20 µM) as inhibitors, and PBS or *E. coli* as activators. Results are given as the area under the curve (AUC), using mean ± standard deviation (n = 6). #; p < 0.05 analyzed using a paired Student’s t-test between the samples with and without *E. coli*, *; p < 0.05 analyzed using one-way ANOVA repeated measurements, Dunnett’s multiple comparisons test, comparing *E. coli*-activated samples with and without inhibitors.

Platelet aggregation during 30 minutes of incubation with heat-inactivated *E. coli* was investigated using Multiplate^®^. *E. coli* significantly increased platelet aggregation from 2 344 to 17 744 AUC (p = 0.0002) (**Figure 1**). Compstatin (Cp40), which inhibits C3 cleavage, reduced the *E. coli*-induced platelet aggregation to 9 947 AUC (44%) (p = 0.0004). No significant effect was seen on platelet aggregation by C5 inhibition with eculizumab (17 542 AUC), CD14 inhibition alone (18 414 AUC), or in combination with compstatin (11 212 AUC). The peptide control for compstatin and the antibody isotype control, did not affect platelet aggregation. The *E. coli* bacteria dose dependently increased platelet aggregation in whole blood (**Figure S1**).

We examined the effect of compstatin on leukocyte-platelet conjugates using flow cytometry. *E. coli* increased the level of both granulocyte-platelet conjugates from 2 531 MFI to 4 263 MFI (p = 0.0012) and monocyte-platelet conjugates from 3 653 MFI to 4 739 MFI (p = 0.0019) (**Figures S2A, B**). Compstatin did not significantly reduce the *E. coli*-induced formation of leukocyte-platelet conjugates.

### *E. coli-*induced platelet aggregation was reduced by C3b inhibition but not by C3a inhibition

**Figure 2:**
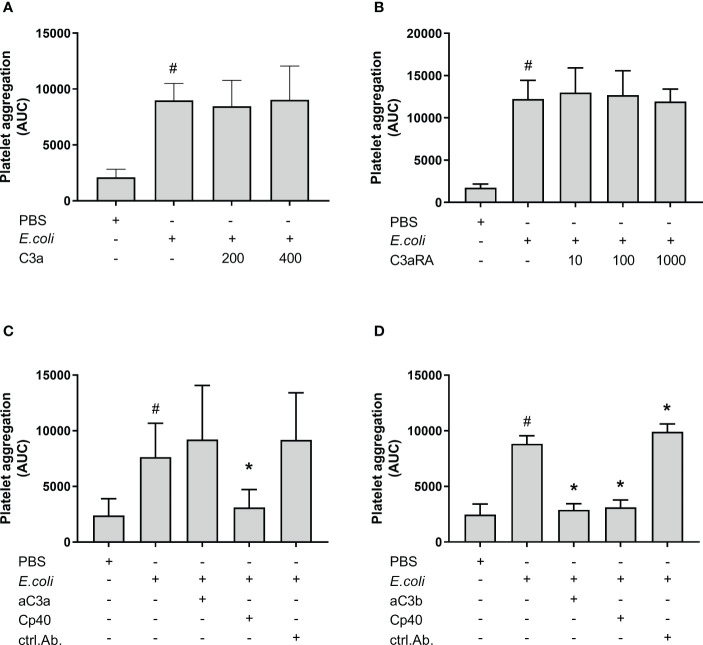
The effect of C3a and C3b (C3a, C3a receptor antagonist (RA), anti-human C3a antibody and blocking anti-human C3b antibody) on *Escherichia coli* (*E. coli* 5.3 x 10^9^/mL)-induced platelet aggregation measured by Multiplate^®^ impedance aggregometry. To the samples were added phosphate-buffered saline (PBS) control or purified C3a in increasing concentrations (200 nM and 400 nM) (n = 5) **(A)**. To the samples were added PBS control or C3aRA in increasing concentrations (10 nM, 100 nM and 1000 nM) (n = 4) **(B)**. To the samples were added PBS control, C3a antibody (aC3a, 0.2 g/mL), compstatin (Cp40, 20 µM) or control antibody (ctrl.Ab, 0.2 mg/mL) (n = 5) **(C)**. To the samples were added PBS control, C3b antibody (aC3b 0.2 mg/mL), compstatin (Cp40, 20 µM) or control antibody (ctrl.Ab 0.2 mg/mL) (n = 3) **(D)**. To all samples, PBS or *E. coli* were added as activators. Results are given as the area under the curve (AUC), using mean ± standard deviation. #; p < 0.05 analyzed using a paired Student’s t-test between the samples with and without *E. coli*, *; p < 0.05 analyzed using one-way ANOVA repeated measurements, and Dunnett’s multiple comparisons test, comparing *E. coli*-activated samples with and without inhibitors or control.

In order to determine the active complement C3 split product(s) responsible for *E. coli*-induced platelet aggregation, we investigated the effect of C3a and C3b (**Figure 2**). *E. coli* significantly increased platelet aggregation from 2 111 AUC to 8 990 AUC (p = 0.0016). The addition of purified C3a in increasing concentrations did not further increase the *E. coli*-induced platelet aggregation (**Figure 2A**). The results were 8 463 AUC and 9 027 AUC, for C3a concentrations of 200 nM and 400 nM.

The C3aRA was added in increasing concentrations without affecting the *E. coli*-induced platelet aggregation (**Figure 2B**). The results were 12 243 AUC, 12 985 AUC, 12 692 AUC and 11 935 AUC for 0 nM, 10 nM, 100 nM and 1000 nM C3aRA, respectively.

The addition of an anti-C3a antibody did not reduce the *E. coli*-induced platelet aggregation, which was 9 206 AUC in contrast to 7 634 AUC for *E. coli* alone (**Figure 2C**). In comparison, compstatin (Cp40) reduced the *E. coli*-induced aggregation by 59% to 3 102 AUC (p = 0.0115).

Collectively, we found that C3a was not responsible for the inhibitory effect of compstatin on the *E. coli*-induced platelet aggregation, and we proceeded with the inhibition of C3b. The anti-C3b blocking antibody significantly reduced the *E. coli*-induced platelet aggregation by 67% from 8 835 AUC to 2 892 AUC (p = 0.0133) (**Figure 2D**), which was in the same range as compstatin, 3 118 AUC (65%) (p = 0.0246). The antibody isotype control did not reduce the aggregation, but rather marginally increased it (p < 0.05) (**Figures 2C, D**).

The complement inhibitory effect of the anti-C3b antibody was determined in the total complement activity assay Wielisa complement system screen (**Figure S3**). As expected, C3 inhibitor compstatin (Cp40) completely blocked all three pathways. In contrast, the anti-C3b antibody blocked only the alternative pathway completely at the concentration used in our experiments (**Figure S3C**), whereas the classical and lectin pathways remained functional to about 50% even in the highest concentrations (**Figures S3A, B**). This is consistent with differential mechanisms for anti-C3b binding to the alternative pathway C3b_n_BbP convertase as compared to the classical and lectin C4bC2bC3b convertases.

### *E. coli* did not induce C3b-opsonization of platelets

**Figure 3:**
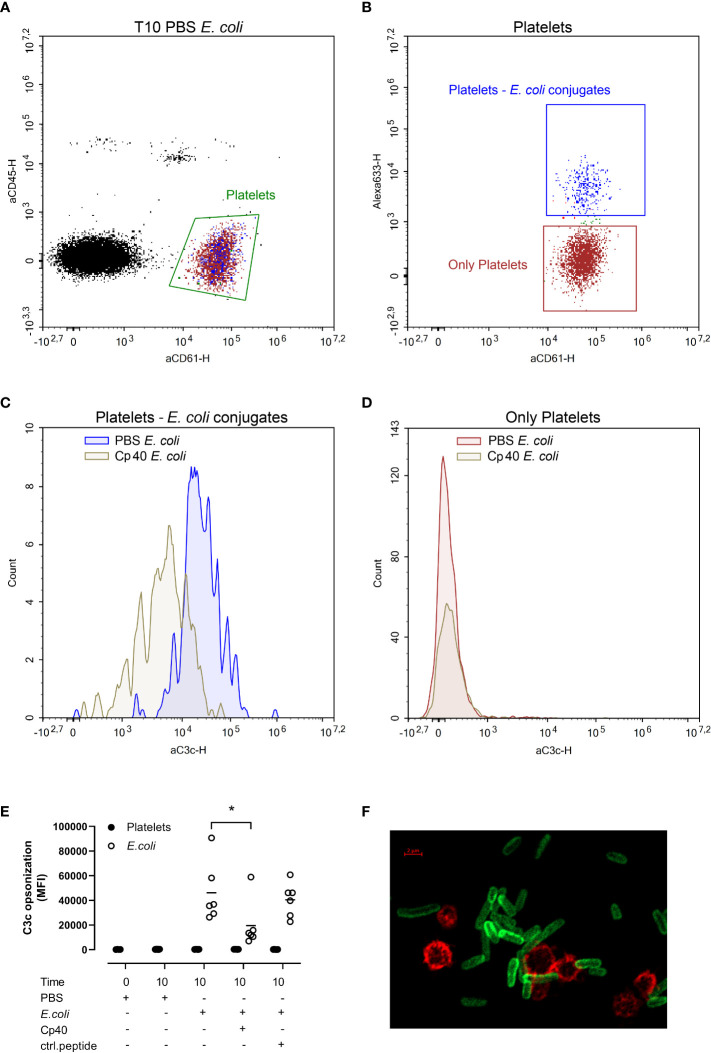
The C3b opsonization of platelets and *Escherichia coli* (*E. coli* 1 x 10^9^/mL) were measured by flow cytometry after performing the human whole blood model. *E. coli* was labeled with Alexa Fluor 633, platelets were detected by BV605-labeled anti-CD61-antibody, leukocytes by V450-labeled anti-CD45-antibody, and C3b opsonization was measured using a FITC-labeled anti-C3c-antibody. The blood was incubated at 37 °C for 10 minutes after adding phosphate-buffered saline (PBS) control, compstatin (Cp40, 20 µM) or control peptide (20 µM), and PBS or *E. coli* as activators. The population of all platelets, both platelets alone and in conjugation with *E. coli*, was gated in an aCD45-aCD61 plot **(A)**. The Alexa 633-aCD61 plot was used to separate the platelets alone (red) and the platelets in conjugation with *E. coli* (blue) **(B)**. Histogram showing the C3b opsonization of platelet-*E. coli* conjugates; the sample without inhibition in blue, the sample with compstatin in gray **(C)**. Histogram showing the C3b opsonization of the platelets alone; the sample without inhibition in red and the sample added compstatin in gray **(D)**. The plots **(A-D)** were from one of the six donors and were representative of the other donors which showed the same pattern. *E. coli*-induced C3b opsonization is shown in a dot plot diagram **(E)**, with data for platelets alone (closed circles) and data for platelets together with *E. coli* (open circles) (n = 6). Results are given in mean fluorescence (MFI) for C3c, the mean is reported as lines in the figure. *; p < 0.05 analyzed using one-way ANOVA repeated measurements, and Dunnett’s multiple comparisons test, comparing *E. coli*-activated samples with and without inhibitors or control. A confocal super-resolution image of platelets interacting with C3b opsonized *E. coli* in whole blood **(F)**. Platelets were detected with a BV605-labeled anti-CD61 antibody (red), and C3b was detected with a FITC-labeled anti-C3c antibody (green).

C3b opsonization of platelets and *E. coli* was measured by flow cytometry (**Figure 3**). We gated the platelets as CD61 positive and CD45 negative cells (**Figure 3A**, green gate). This population was further gated based on the *E. coli-*conjugated Alexa Fluor 633 to obtain two populations; platelets without *E. coli*, and *E. coli*-bound platelets (**Figure 3B**). C3b opsonization of platelet-*E. coli* conjugates were significantly reduced in samples containing compstatin (Cp40) (p < 0.05) (**Figures 3C, E**). In contrast, platelets alone were unaffected by compstatin (Cp40) (**Figures 3D, E**). The results are summarized in **Figure 3E**. Compstatin (Cp40) significantly reduced the C3c opsonization of *E. coli* by 57% from 46 165 MFI in the samples without inhibition to 19 623 MFI (p = 0.0187). With confocal microscopy, we confirmed that *E. coli* was opsonized by C3b, but platelets were not (**Figure 3F** and **Figure S4A**). An isotype control antibody showed no binding (**Figure S4B**, **C**).

### Platelet aggregation was higher in hirudin whole blood compared to hirudin PRP

**Figure 4:**
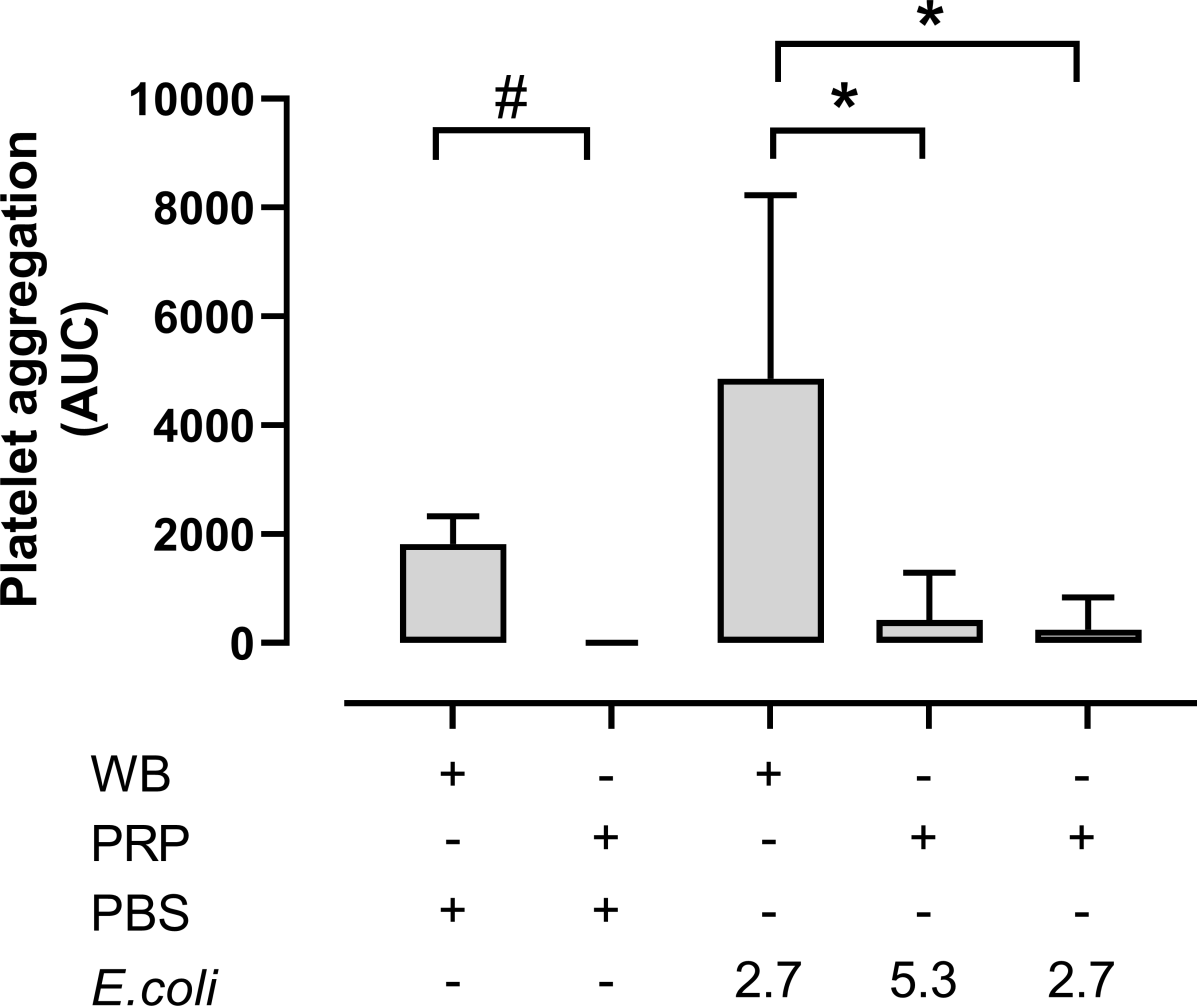
The platelet aggregation was performed in both hirudin whole blood (WB) and hirudin platelet-rich plasma (PRP). The samples were analyzed with phosphate-buffered saline (PBS) or *Escherichia coli* (*E. coli* 2.7 x 10^9^/mL or 5.3 x 10^9/^mL) on Multiplate^®^ impedance aggregometry (n = 6). Results are given as the area under the curve (AUC), using mean ± standard deviation. #; p < 0.05 analyzed using a Wilcoxon test between the whole blood samples and PRP without *E. coli*, *; p < 0.05 analyzed using a Friedman test and Dunn’s multiple comparisons test, comparing *E. coli*-activated whole blood samples with *E. coli*-activated PRP.

Platelet aggregation in whole blood versus PRP was then compared (**Figure 4**). Notably, platelet aggregation in hirudin PRP was significantly lower than in hirudin blood both in the absence and the presence of *E. coli*. Without *E. coli*, platelet aggregation in whole blood was 1820 AUC, while it was hardly detectable in PRP (p = 0.0313). The *E. coli*-induced platelet aggregation was 4857 AUC in whole blood (2.7 x 10^9^ *E. coli*/mL) and 423 AUC and 244 AUC in the PRP samples with 5.3 x 10^9^/mL and 2.7 x 10^9^ *E. coli*/mL, respectively (p = 0.0078 and p = 0.0418).

### *E. coli* did not induce aggregation of isolated platelets

**Figure 5:**
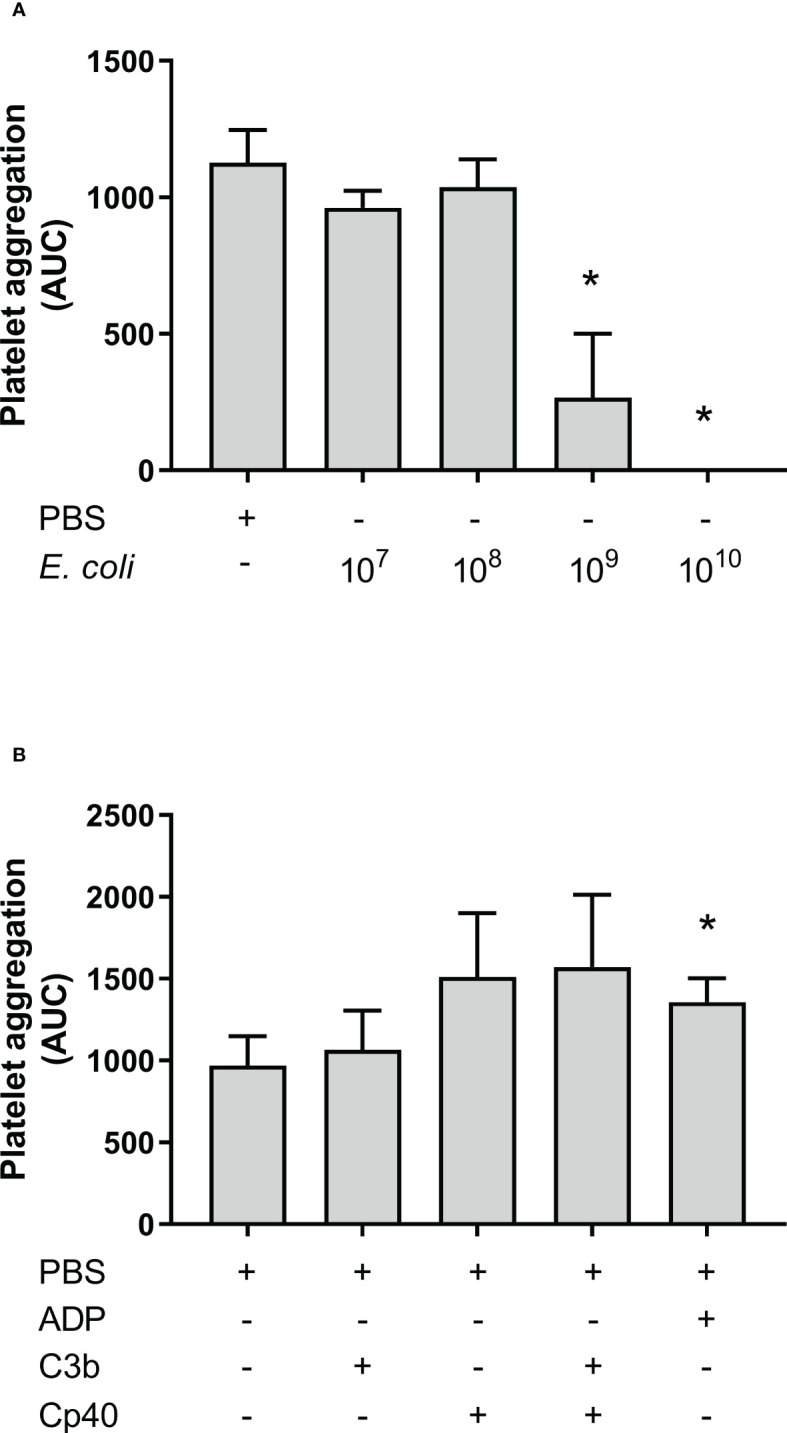
Platelet aggregation with isolated platelets in Tyrode’s buffer was measured by Multiplate^®^ impedance aggregometry. The platelets were isolated by centrifugation and washed using Tyrode’s buffer. The samples were added phosphate-buffered saline (PBS) or *Escherichia coli* (*E. coli*) in PBS in increasing concentrations as indicated; 1 x 10^7^/mL, 1 x 10^8^/mL, 1 x 10^9^/mL, and 1 x 10^10^/mL, n = 3 **(A)**. *; p < 0.05 analyzed using one-way repeated measurements ANOVA, and Dunnett’s multiple comparisons test, comparing samples with increasing *E. coli* concentrations to the lowest *E. coli* concentration (1 x 10^7^/mL). The effect of C3b on aggregation of isolated platelets were analyzed **(B)**. To the samples were either added PBS, C3b (20 nM), compstatin (Cp40, 20 µM) or a combination of them. Adenosine diphosphate (ADP) was added as a positive control. The results are given as the area under the curve (AUC), using mean ± standard deviation (n = 5). *; p < 0.05 analyzed using one-way ANOVA repeated measurements, and Dunnett’s multiple comparisons test, comparing the PBS control to the other samples.

Aggregation of isolated platelets in buffer was measured by Multiplate^®^. *E. coli* was added at increasing concentrations to isolated platelets but this did not increase platelet aggregation compared to PBS. Instead, the highest *E. coli* concentration significantly inhibited platelet aggregation (**Figure 5A**), from 962 AUC at 1 x 10^7^/mL to 267 AUC at 1 x 10^9^/mL (p = 0.0374), and no platelet aggregation was detected using 1 x 10^10^/mL *E. coli* (p = 0.0028). Since *E. coli* did not induce aggregation of isolated platelets, we explored the effect of externally added purified C3b on platelets in the presence and absence of compstatin. Spontaneous platelet aggregation in isolated platelets was 970 AUC without and 1 065 AUC with C3b, respectively (p = 0.4133), indicating no effect of the presence of C3b (**Figure 5B**). Compstatin did not affect the aggregation of isolated platelets. ADP was included as a positive control for platelet aggregation (p = 0.0011).

### The *E. coli*-induced platelet aggregation and inhibition of CR1

**Figure 6:**
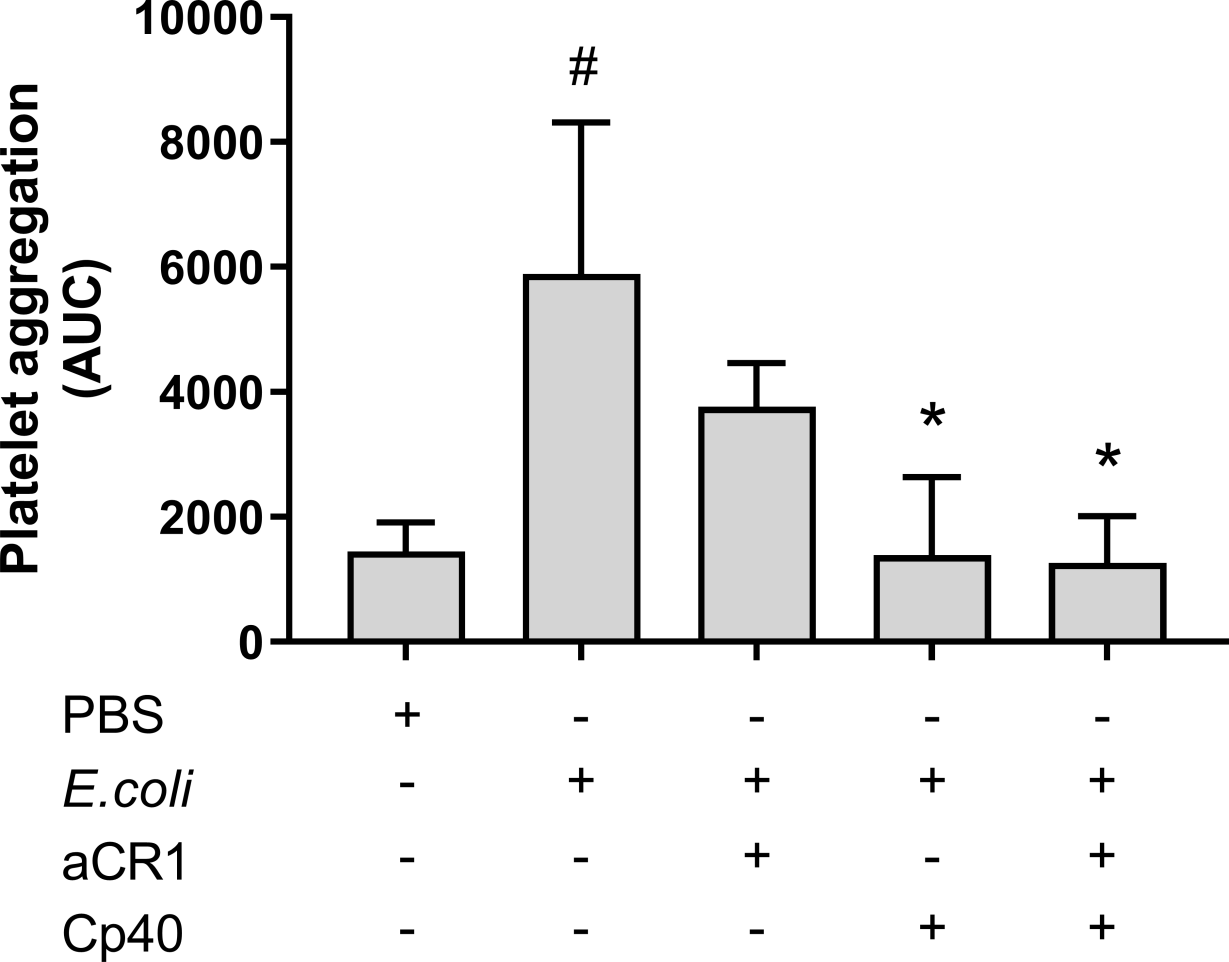
The effect of a blocking complement receptor (CR)1 antibody on *Escherichia coli* (*E. coli* 5.3 x 10^9^/mL)-induced platelet aggregation measured on Multiplate^®^ impedance aggregometry. To the samples were added phosphate-buffered saline (PBS) control, compstatin (Cp40, 20 µM), blocking CR1 antibody (4 ng/mL) or a combination of both, and PBS or *E. coli* as activators. Results are given as the area under the curve (AUC), using mean ± standard deviation (n = 7). #; p < 0.05 analyzed using a paired Student’s t-test between the samples with and without *E. coli*, *; p < 0.05 analyzed using one-way ANOVA repeated measurements, and Dunnett’s multiple comparisons test, comparing *E. coli*-activated samples with and without inhibitors.

To determine whether C3b-dependent platelet aggregation involves CR1, a blocking anti-CR1 antibody was included in the Multiplate^®^ experiment. The anti-CR1 antibody reduced the *E. coli*-induced platelet aggregation from 5 890 to 3 760 AUC (36%), although not significant, a statistical trend was found (p = 0.054) (**Figure 6**). The combined inhibition of CR1 and C3 with compstatin (Cp40) did not reduce platelet aggregation more than compstatin alone, resulting in 1 388 AUC (76%) compared to 1 261 AUC (79%), respectively.

### Complement C3 and the GPIIb/IIIa receptors inhibition revealed additive effects on the *E. coli*-induced platelet aggregation

**Figure 7:**
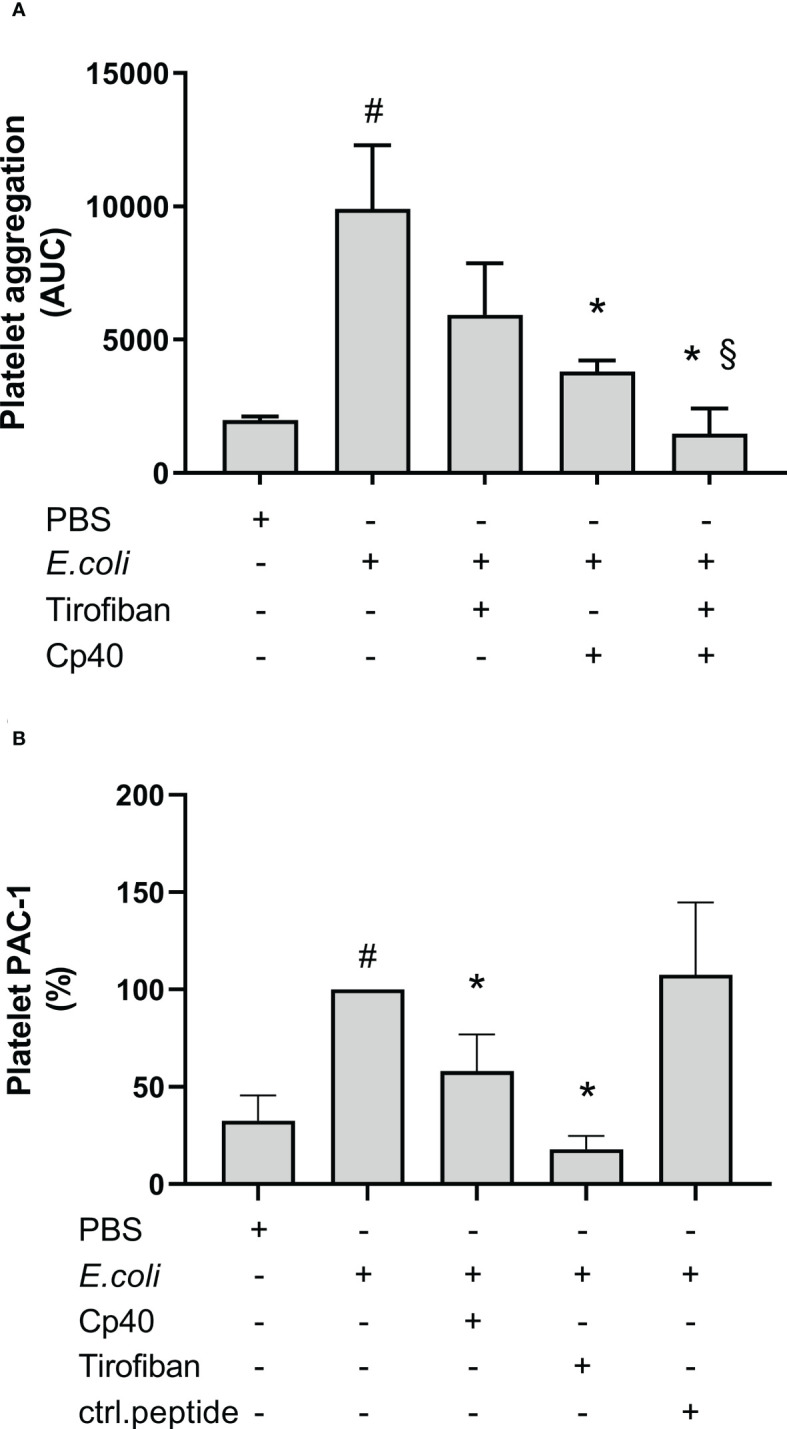
The role of glycoprotein (GP) IIb/IIIa on *Escherichia coli* (*E. coli* 5.3 x 10^9^/mL)-induced platelet aggregation measured by Multiplate^®^ impedance aggregometry **(A)**. To the samples were added phosphate-buffered saline (PBS) control, tirofiban (5 µg/mL), compstatin (Cp40, 20 µM) or the combination of tirofiban and compstatin, and PBS or *E. coli* as activators. The results are given as the area under the curve (AUC), using median ± interquartile range (n = 6). The effect of C3-inhibition on the *E. coli*-induced (1 x 10^9^/mL) activated GPIIb/IIIa on platelets measured by flow cytometry **(B)**. To the samples were added PBS control, tirofiban (5 µg/mL), compstatin (Cp40, 20 µM), or control peptide (ctrl.peptide, 20 µM), and PBS or *E. coli* as activators. A BV605-labeled anti-human CD61 antibody was used to gate the platelets and a FITC-labeled anti-human PAC-1 antibody was used to detect the activated GPIIb/IIIa receptors. Results are expressed as percent of the uninhibited sample (*E. coli* without inhibition set to 100%) and are given as median ± interquartile range (n = 16). #; p < 0.05 analyzed using a Wilcoxon test between the samples with and without *E. coli*, *; p < 0.05 analyzed using a Friedman test and Dunn’s multiple comparisons test, comparing *E. coli*-activated samples without and with inhibition or control. §; p < 0.05 analyzed using a Wilcoxon test between the *E. coli*-activated samples with compstatin (Cp40) and with the combination of compstatin and tirofiban.

The GPIIb/IIIa receptor inhibitor, tirofiban, non-significantly reduced the *E. coli*-induced platelet aggregation by 40% from 9 905 AUC to 5 931 AUC measured by Multiplate® (**Figure 7A**). In comparison, compstatin (Cp40) significantly reduced the platelet aggregation by 62% to 3 801 AUC (p = 0.0417). Notably, the combination of both inhibitors had an even more profound effect (p = 0.0313) than compstatin alone and significantly reduced the *E. coli*-induced platelet aggregation by 85% to 1 471 AUC (p = 0.0002), which is below the background level (1 980 AUC). These results indicated that the GPIIb/IIIa receptor is involved in *E. coli*-induced platelet aggregation, in addition to C3b.

### The *E. coli*-induced activation of GPIIb/IIIa receptor was partially C3-dependent

GPIIb/IIIa receptor activation was measured using flow cytometry and the monoclonal anti-PAC-1 antibody, which specifically binds to the activated form of GPIIb/IIIa*. E. coli* significantly increased this binding by 207% compared to PBS (p < 0.0001) (**Figure 7B**). Compstatin (Cp40) significantly reduced the GPIIb/IIIa upregulation by 42% (p < 0.0001), which indicated that GPIIb/IIIa receptor activation was partly C3-dependent. As a control, the specific GPIIb/IIIa inhibitor tirofiban reduced the activation by 82% (p < 0.0001), which is below the background level. In comparison, the control peptide did not affect the *E. coli*-induced activation of GPIIb/IIIa (p > 0.05).

### The *E. coli*-induced platelet aggregation was partly P-selectin-dependent

P-selectin has been shown to bind C3b (26), in addition to P-selectin glycoprotein ligand-1. Therefore, we wanted to determine if P-selectin was involved in the C3-dependent platelet aggregation. The *E. coli*-induced platelet aggregation was slightly lower (24%) when P-selectin was blocked; 8 135 AUC vs 10 652 in the uninhibited sample, but the difference was not statistically significant (**Figure S5**). As a positive inhibition control, compstatin (Cp40) again significantly reduced the *E.coli*-induced platelet aggregation by 59% to 4 317 AUC (p = 0.0006). The control peptide did not affect platelet aggregation, which was 10 431 AUC.

### C3a did not increase ADP-induced platelet aggregation

**Figure 8:**
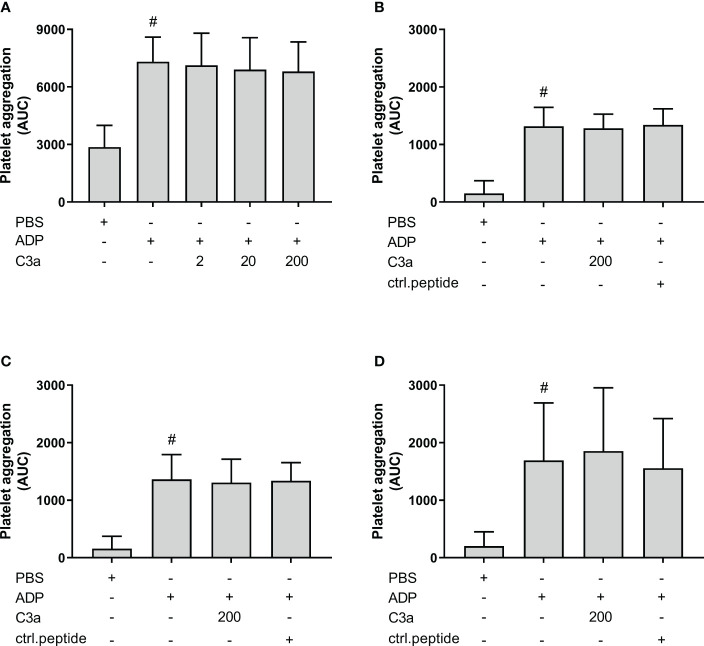
The effect of C3a addition on adenosine diphosphate (ADP)-induced platelet aggregation measured on Multiplate^®^ impedance aggregometry with whole blood and platelet-rich plasma (PRP) in different anticoagulants. To hirudin whole blood was added phosphate-buffered saline (PBS) control, ADP (2 µM), and C3a (2, 20 and 200 nM) (n = 6) (**A**). Hirudin PRP with PBS control, ADP (2 µM), C3a (200 nM) or control peptide (200 nM) (n = 4) (**B**). ACD PRP with PBS control, ADP (2 µM), C3a (200 nM) or control peptide (ctrl.peptide 200 nM) (n = 6) (**C**). Citrate PRP with PBS control, ADP (2 µM), C3a (200 nM) or control peptide (200 nM) (n = 6) (**D**). Results are given as the area under the curve (AUC), using mean ± standard deviation. #; p < 0.05 analyzed using a paired Student’s t-test between the samples with and without ADP, and Dunnett’s multiple comparisons test, comparing ADP-activated samples with and without inhibitors or control.

Others have reported that C3a increased ADP-induced platelet aggregation using isolated platelets. In order to investigate the effect of C3a in PRP samples, we included experiments in both hirudin whole blood and PRP, ACD PRP and citrate PRP (**Figures 8A–D**). ADP significantly increased the platelet aggregation in comparison to PBS in all samples (p < 0.05). However, C3a addition did not further increase the ADP-induced platelet aggregation in whole blood or PRP.

### *E. coli* is a strong agonist for platelet aggregation

To evaluate the effect of *E. coli* as an activator of platelet aggregation in relation to ADP, blood from six donors were analyzed by Multiplate^®^ (**Figures S6A, B**). *E. coli* (5.3 x 10^9^/mL) increased the platelet aggregation by 528% from 2 392 AUC to 12 650 AUC, while ADP (2.1 µM) increased the platelet aggregation by 232% to 5 547 AUC. *E. coli* (5.3 x 10^9^/mL) was a significantly stronger agonist than ADP (2.1 µM) (p = 0.0026).

### The release of soluble platelet activation markers P-selectin and βTG was not complement-dependent

In the whole blood model, *E. coli* significantly increased the plasma levels of P-selectin and βTG from 37.5 to 63.7 ng/mL (70% increase) and from 5 156 to 14 052 pg/mL (172%), respectively (p = 0.0003 and p = 0.0033) (**Figure S7**). Inhibition of complement at both the C3 by compstatin (Cp40) and the C5/C5aR level using eculizumab and PMX53 alone or in combination with a blocking anti-CD14 antibody did not reduce the *E. coli*-induced release of P-selectin and βTG, indicating complement-independent mechanisms for the platelet release reaction in contrast to platelet aggregation.

## Discussion

We found that the *E. coli*-induced platelet aggregation was complement-dependent at the C3-level, and the data support C3b as the main responsible fragment. Notably, we observed that *E. coli*, but not platelets, were opsonized by C3b. Complement inhibition at the level of C3 blocked C3b opsonization of *E. coli* as well as platelet aggregation. *E. coli*-induced platelet aggregation seemed to be partially dependent on the two receptors, CR1 and GPIIb/IIIa. The activation of GPIIb/IIIa was partly complement-dependent since compstatin significantly reduced the binding of the anti-PAC-1 antibody. Accordingly, this C3-dependent activation could explain that the combined effect of tirofiban and compstatin reduced the *E. coli*-induced platelet aggregation even more than single inhibition. Notably, the platelet release reaction, as measured by soluble P-selectin and βTG, was complement-independent.

Compstatin inhibits the cleavage of native C3 to its active fragments C3a and C3b. Thus, we aimed to further analyze the C3 dependence of *E. coli*-induced platelet aggregation in human whole blood. Importantly and surprisingly we observed that the anaphylatoxin C3a, a C3aR antagonist, and a blocking anti-C3a antibody did not affect the platelet aggregation. In contrast, a blocking anti-C3b antibody reduced *E. coli*-induced platelet aggregation significantly, and we concluded that the effect of compstatin was due to the reduced level of C3b, and not C3a. In order to exclude the possibility that compstatin and the anti-C3b antibody worked by the same inhibitory mechanisms, we performed a Wielisa complement system screen. The anti-C3b antibody blocked the alternative pathway completely; however, the classical and lectin pathway remained active, albeit at reduced levels.

A baboon model with *E. coli*-induced sepsis showed that C3 inhibition by compstatin reduced platelet aggregation, similar to our *in vitro* model (40). This was shown by measuring reduced platelet accumulation in the lungs and less C5b-9 deposition on platelet aggregates. C3(H_2_O) has been shown to bind TRAP-6-activated platelets (41). We examined the C3b opsonization of platelets and *E. coli* using flow cytometry and confocal microscopy. C3b-fragments could not be detected on platelets after incubation with *E. coli* for 10 minutes, possibly due to absent thrombin-induced PAR-1 activation in lepirudin anticoagulated whole blood. However, the bacteria showed a high degree of C3b opsonization.

The platelet aggregation was higher in hirudin anticoagulated whole blood compared to PRP activated with *E. coli*. The interaction with other cell types in whole blood is most likely promoting platelet aggregation in samples without thrombin-mediated activation of the platelets, as is the case in blood with lepirudin or hirudin, which blocks thrombin. However, when applying citrate as an anticoagulant, the difference between whole blood and PRP was minor, and some blood donors had a higher aggregation in PRP than in whole blood (data not shown). This can indicate two different explanations; 1) aggregation without the presence of thrombin, in the hirudin tubes, is dependent on the interaction with other cells, in contrast to platelet aggregation, which does not rely on interaction with other cells in the citrate tubes. Erythrocytes did not activate platelets or increase the aggregation in citrated blood (42). 2) In citrate anticoagulated samples there is some residual free calcium that supports the activation of the GPIIb/IIIa receptor (43). We speculate that *E. coli*-activation induces dense granules release, and accompanied by other mechanisms, facilitates the coagulation process and thrombin-induced activation of the platelets in citrated blood.

We also investigated the effect of *E. coli* on isolated platelets in Tyrode’s buffer. *E. coli* did not induce platelet aggregation which confirmed that the *E. coli*-induced platelet aggregation in hirudin blood was dependent on other components present in the whole blood. We hypothesized that C3b was essential for complete *E. coli*-induced platelet aggregation and therefore proceeded by adding C3b to isolated platelets in the absence of other complement components. However, no effect was observed from the purified C3b. The addition of compstatin induced a slight increase in platelet aggregation but this increase was not significant. This indicates that *E. coli* and C3b alone in solution were not sufficient to induce platelet aggregation in the absence of plasma proteins and other cells.

The erythrocytes have a high expression of CR1 (44). CR1 can bind to C3b (45) and could be a possible link between C3b bound to *E. coli* and erythrocytes. Erythrocyte CR1 binds 80-90% of *E. coli* added in the whole blood model (39). Thus, we investigated the role of CR1 in *E. coli*-induced platelet aggregation. We targeted this receptor by using a specific blocking antibody, which inhibits the binding of C3b opsonized bacteria to erythrocytes (36, 39). *E. coli-*induced platelet aggregation was 36% lower in the presence of the antibody, but the difference was only a statistical trend (p = 0.054). A type II statistical error is most likely, and we can therefore not exclude that CR1 is partly involved in the *E. coli*-induced platelet aggregation. The combined inhibition of CR1 and C3 with compstatin did not reduce platelet aggregation more than compstatin alone, indicating that C3b is mainly responsible for these effects. Since the reduction induced by the anti-CR1antibody was minor compared to compstatin, we suggest that the mechanism involves several other interactions. The erythrocytes express other receptors and can also bind fibrinogen that links erythrocytes together (46). A previous study showed that erythrocytes increased both the activation of GPIIb/IIIa on platelets and P-selectin levels (47), confirming that the interaction between erythrocytes and platelets intensifies platelet aggregability.

Fibrinogen connects platelets by binding to several GPIIb/IIIa receptor molecules. We used tirofiban, a GPIIb/IIIa inhibitor, and showed that this receptor is involved in *E. coli*-induced platelet aggregation. Interestingly, the combined inhibition of GPIIb/IIIa and C3, with tirofiban and compstatin, abolished the *E. coli*-induced platelet aggregation. C3 has been shown to bind fibrinogen and stabilize the clot by prolonging fibrinolysis, linking GPIIb/IIIa, fibrinogen, and C3 to thrombosis risk (48). The activation of the GPIIb/IIIa is calcium-dependent (49) and can be induced by ADP, thrombin or other agonists (50). To the best of our knowledge, an interaction between the activation of this receptor and the complement system is unknown. Our flow cytometry analysis showed that compstatin inhibited the activation of the GPIIb/IIIa receptor on platelets, indicating that there is a link between GPIIb/IIIa and C3. It has been shown that the IgG receptor FcγRIIa on platelets has a role in *E. coli*-induced platelet aggregation (51). IgG against the outer membrane proteins of *E. coli* is naturally occurring in healthy human plasma (52, 53). Engagement of the GPIIb/IIIa receptor in the presence of IgG is necessary for FcγRIIa phosphorylation and dense granula release (51). We therefore hypothesis that C3b has a role in activation of the FcγRIIa receptor through activation of the GPIIb/IIIa receptor. Further studies are needed to determine the exact role of C3 in GPIIb/IIIa activation.

C3b may also influence the FcγRIIa by opsonizing the immune complexes. C3b can bind covalently to the Fab and Fc-region of IgGs and thereby induce alternative pathway activation (54, 55). Immune complexes have a higher affinity to FcγR than monomeric IgG (56). By using compstatin, the generation of C3b is blocked. The lack of C3b on IgG might reduce the engagement of the CR1 on red blood cells. We hypothesize that this mechanism could partly explain the effect of compstatin and the anti-CR1 antibody on platelet aggregation in whole blood in this study.

In this study, we have shown that the *E. coli*-induced platelet aggregation was partly C3b-dependent. A previous study reported that C3b bound to TRAP-6-activated platelets via P-selectin, and initiated the complement cascade (26). They found that treatment with proteinase K, removing all surface proteins, abolished the C3b binding to isolated platelets (26). This indicated that C3b does not bind directly to the platelet membrane but through a receptor. Since we found that C3b is an important molecule in *E. coli*-induced platelet aggregation, we wanted to test if P-selectin could bind C3b exposed on *E. coli* and mediate platelet aggregation. Inhibition by a P-selectin-specific antibody only slightly and non-significantly reduced the *E. coli*-induced platelet aggregation. We could neither confirm nor disprove the effect of P-selectin on platelet aggregation using our human whole blood model.

Activated platelets make conjugates with leukocytes, and we examined the C3-dependence of these interactions. Compstatin did neither reduce the *E. coli*-induced granulocyte-platelet nor monocyte-platelet conjugates, as seen in platelet-platelet conjugates. The effect of compstatin on platelet aggregation could therefore not be explained by changes in the leukocyte-platelet conjugates. However, we suggest that the interaction with other blood cells is necessary for *E. coli*-induced platelet aggregation as discussed earlier.

Importantly, although we have shown that *E. coli*-induced platelet aggregation was at least partly complement C3b-dependent, we did not find complement dependency for platelet activation in our model when measuring the soluble platelet activation markers P-selectin and βTG by ELISA. However, an *in vivo* study found that compstatin reduced both platelet aggregation and activation, measured by decreased plasma levels of P-selectin (40). Although the human whole blood model is mimicking the physiologic situation it does not fully represent the *in vivo* condition. In addition, the whole blood model used lacks endothelial cells. Thus, as for all *in vitro* and *ex vivo* studies, the results from the present study should be interpreted with caution concerning the translational value to human physiology and clinical medicine.

Earlier studies have indicated that the connection between complement and platelet aggregation was C3a-driven; Polley and Nachman reported that C3a increased the ADP-induced aggregation of platelets (15), and Sauter et al. later confirmed these findings in a similar study (16). Both worked with filtered and isolated human platelets kept in a buffer and applied ADP as the platelet activator. In contrast, we performed experiments in human whole blood with *E. coli*-induced platelet aggregation. There are several advantages to using a whole blood model when studying platelets; they have an important role in intercellular communication, extracellular signaling through receptors, and transfer of surface molecules, which all are mechanisms that are interrupted or altered when platelets are removed from their natural surroundings in blood. Furthermore, platelets might be activated and present as a different phenotype in purified platelet solutions than in native human whole blood. These factors might explain the apparent contradiction between the previous C3a findings and our findings supporting C3b as the main player. We argue that we applied a more physiologically relevant human whole blood model compared to isolated platelets.

The mechanism of platelet aggregation is also strongly dependent on the agonist. The studies mentioned above used ADP as a platelet activator (15, 16). We, therefore, tested the effect of C3a on ADP-induced platelet aggregation with our system in both whole blood and PRP, and performed the experiments as earlier, with preincubation with ADP before the addition of C3a. We did not find any increased platelet aggregation in hirudin whole blood, hirudin PRP, citrate PRP or ACD PRP, when C3a was added at concentrations of up to 200 nM. To exclude the possibility that a C3a effect was hidden as a result of preincubation with ADP, we also added C3a and ADP simultaneously in hirudin whole blood. However, no differences between the two setups could be seen (data not shown). The primary ADP-induced platelet aggregation pathway relies mainly on the activation of two platelet P2Y receptors, which again induce the secretion of ADP from dense granules. This pathway is different from the *E. coli*-induced platelet aggregation, where we hypothesize that C3b, and not C3a, plays a central role. In conclusion, we could not confirm the earlier studies showing a C3a-dependent platelet aggregation using isolated platelets.

There are some limitations of this study; the use of lepirudin as an anticoagulant excludes us from studying the effects of thrombin in platelet aggregation. The Multiplate^®^ Analyzer is designed to test platelet function in whole blood. Test results using PRP and isolated platelets must therefore be interpreted with caution. There are several platelet receptors that have not been examined in this study, and this will limit our conclusions. As mentioned above, an *in vitro* study is not equivalent to studying live organisms. Despite these limitations, our platelet aggregation model enables us to study interactions between platelets, plasma proteins, and other blood cells in whole blood. We titrated *E. coli* concentration between 10^7^-10^10^ per mL and continued with 10^9^ per mL, the concentration that yielded the highest degree of platelet aggregation. This concentration is higher than what is seen in most cases of sepsis, although comparable bacteria loads have been reported previously in humans with sepsis (57).

## Conclusions

In this study, we have shown that the *E. coli*-induced platelet aggregation in human whole blood is partly, but still substantially, dependent on C3b, but not dependent on C3a. Other mechanisms and mediators contributing to this aggregation might include activation of the GPIIb/IIIa receptor, CR1 and P-selectin.

## Data availability statement

The original contributions presented in the study are included in the article/**Supplementary Material**. Further inquiries can be directed to the corresponding author.

## Ethics statement

The studies involving human participants were reviewed and approved by the regional ethics committee in Northern Norway Regional Healthy Authority. The patients/participants provided their written informed consent to participate in this study.

## Author contributions

AL, ÅE, PN, and O-LB designed the study. AL, ÅE, and AG performed the experiments. AL and ÅE wrote the manuscript. AL did the statistical analysis. BK took the confocal pictures. CL and DC gave advice with the flow cytometry analysis and Wielisa. TM, PN, and O-LB gave advice throughout the study. CL, AG, TM, PN, and O-LB revised the manuscript. All the authors have read, commented and approved the manuscript.
