# Supplementary material for: Complement C3b contributes to Escherichia coli-induced platelet aggregation in human whole blood
Source: Front Immunol. 2022 Dec 14;13:1020712. doi: 10.3389/fimmu.2022.1020712 (PMC9797026; doi:10.3389/fimmu.2022.1020712)
Supplement: Supplementary file 1 [file DataSheet_1.pdf]

Supplementary Material

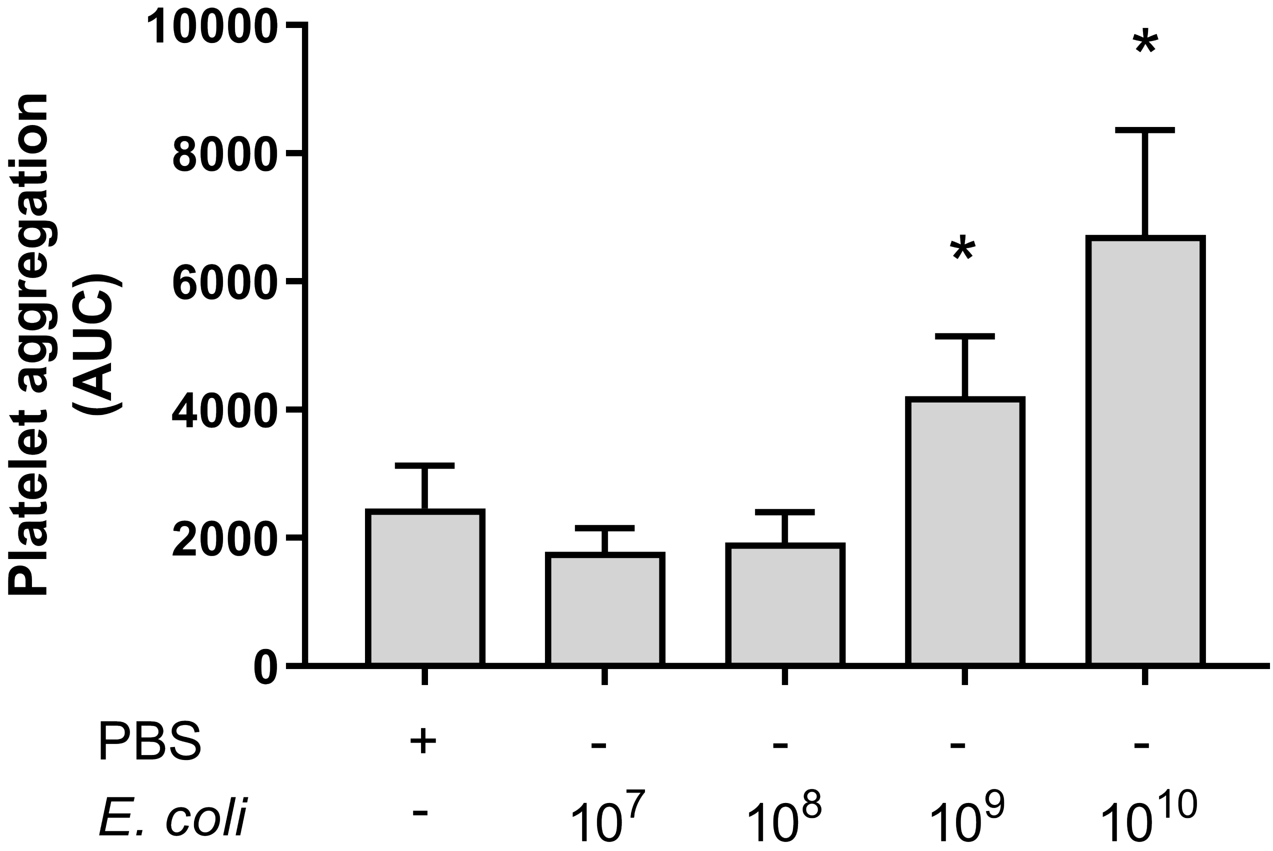

Supplementary Figure 1. The effect of increasing *Escherichia coli* (*E. coli*) concentrations (1 x 10^7/mL to 1 x 10^{10}/mL) on platelet aggregation was analyzed by Multiplate® impedance aggregometry. Results are given as the area under the curve (AUC), using mean ± standard deviation (n = 4). *; $p < 0.05$ analyzed using one-way repeated measurements ANOVA, and Dunnett's multiple comparisons test, comparing samples with increasing *E. coli* concentrations to the lowest *E. coli* concentration (1 x 10^7/mL).

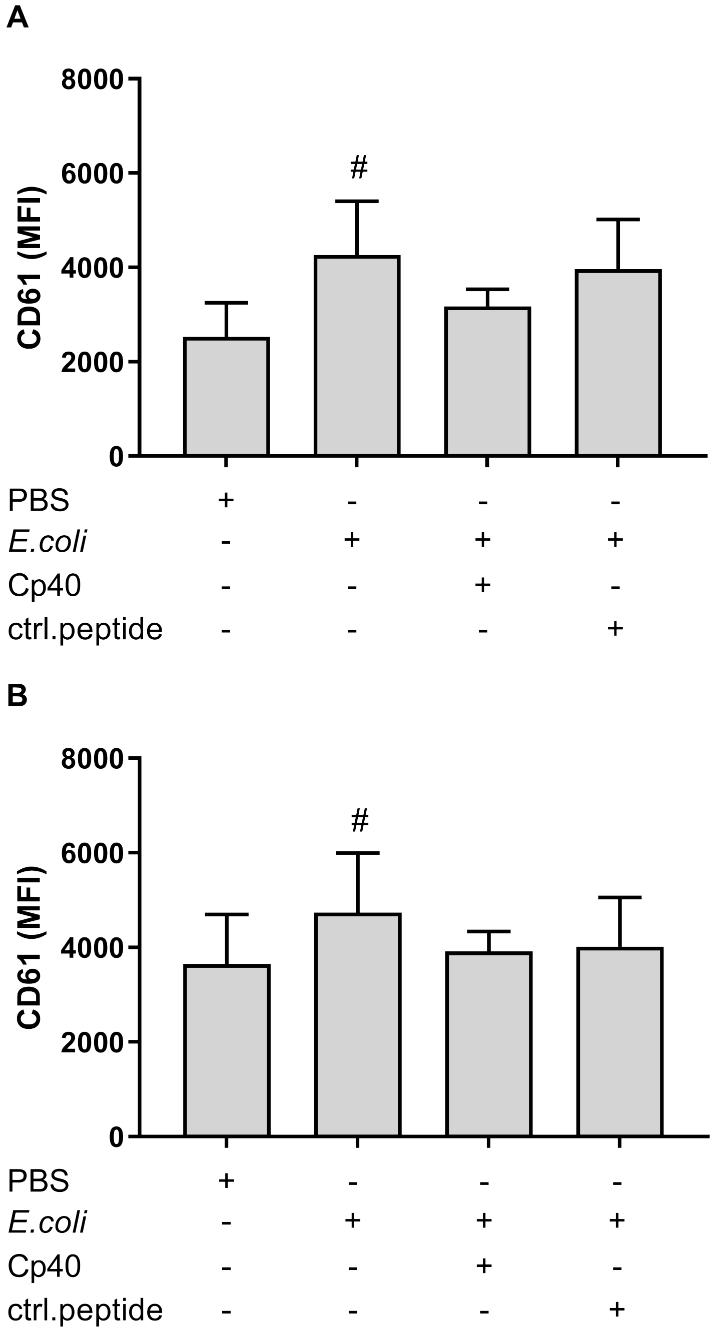

Supplementary Figure 2. *Escherichia coli* (*E. coli*, 5 x 10^8/mL)-induced granulocyte-platelet conjugates (**A**) and monocyte-platelet conjugates (**B**) were analyzed using flow cytometry. The platelets were stained by a FITC-labeled anti-CD61-antibody, leukocytes by a V500-labeled anti-CD45-antibody, granulocytes by a BV650-labeled anti-CD15-antibody and monocytes by an Alexa Fluor 647-labeled anti-CD14-antibody. The blood was incubated at 37 ºC for 15 minutes after adding phosphate-buffered saline (PBS) control, compstatin (Cp40, 20 µM) or control peptide (ctrl. peptide, 20 µM), and PBS or *E. coli* as activators (n = 6). Results are given in mean fluorescence intensity (MFI) for anti-CD61-antibody. #; $p < 0.05$ analyzed using a paired Student's t-test between the samples with PBS and *E. coli*.

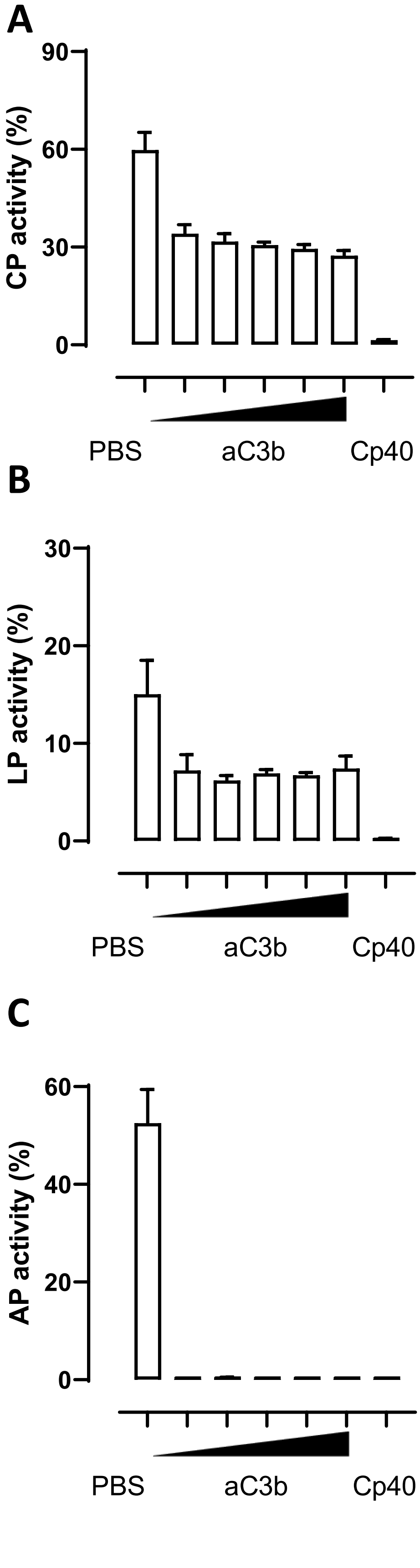

Supplementary Figure 3. A Wielisa test was performed in order to determine the effect of the C3b-antibody on the three complement activation pathways, classical pathway (CP) (**A**), lectin pathway (LP) (**B**) and alternative pathway (AP) (**C**). The serum samples were added phosphate-buffered saline (PBS) control, several concentrations of C3b antibody 0.28, 0.43, 0.58, 0.86, 1.15 mg/mL or compstatin (Cp40, 20 µM) and analyzed in duplicates. The concentration 0.58 mg/mL of the blocking anti-C3b antibody in this test was equivalent to the concentration used in the Multiplate® C3b experiments. The activity of the positive control was set to 100% and the results are given in mean percent ± standard deviation (n = 2).

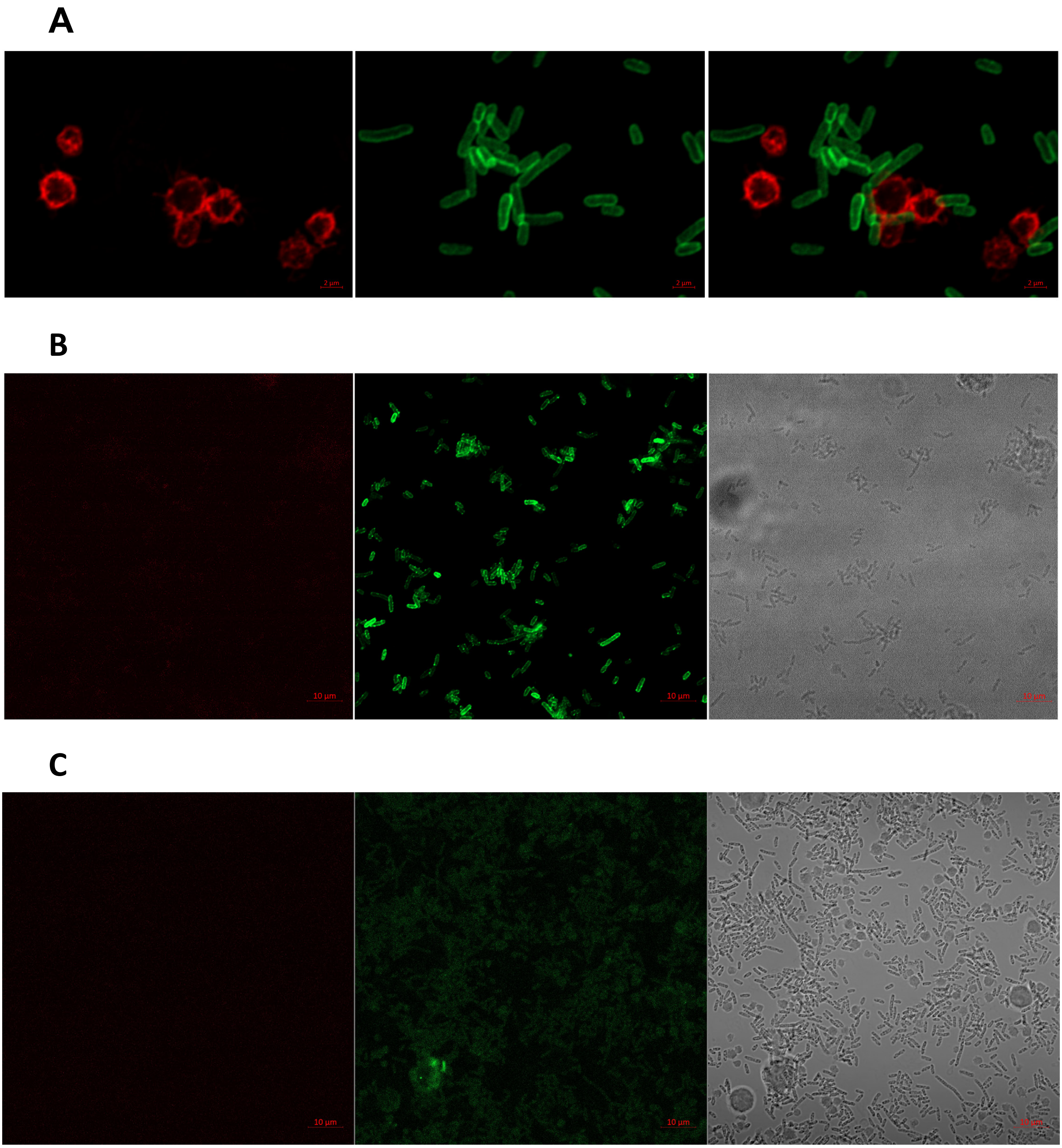

Supplementary Figure 4. Super-resolution image of platelets interacting with C3b-opsonized *Escherichia coli* (*E. coli*) in whole blood (**A**). Platelets were detected with a BV605-labeled anti-CD61 antibody, and C3b was detected by a FITC-labeled anti-C3c antibody. Combined confocal and differential interference contrast (DIC) image of C3b-opsonized *E. coli* in whole blood (**B**). C3b was detected by a FITC-labeled anti-C3c antibody. Platelets were unlabeled. Combined confocal and DIC image of *E. coli* in whole blood after applying a FITC-labeled polyclonal rabbit anti-mouse antibody (**C**). This antibody was used as a negative control to verify the specificity of the FITC-labeled anti-C3c antibody. Platelets were unlabeled.

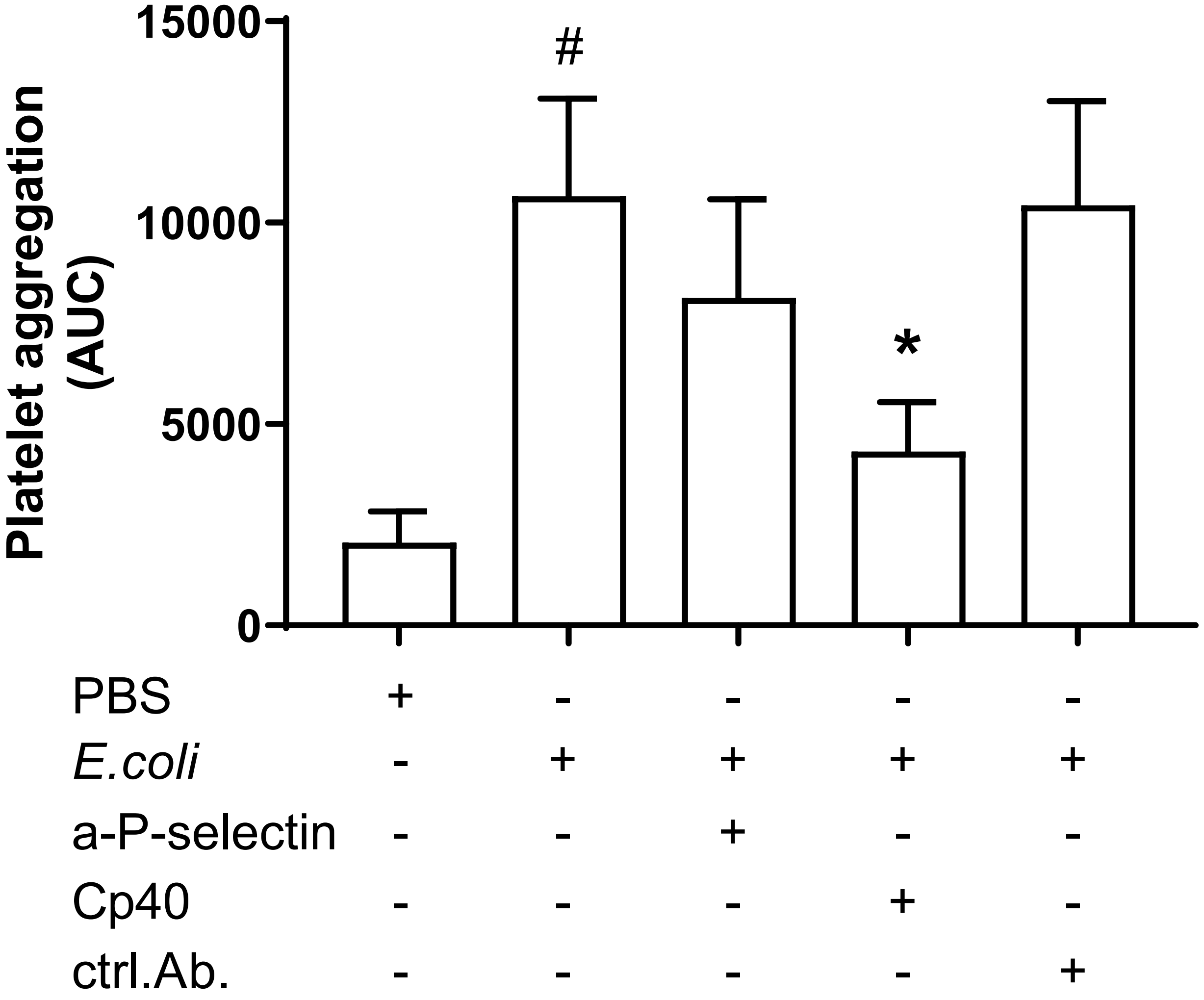

Supplementary Figure 5. The effect of anti-human P-selectin antibody on *Escherichia coli* (*E. coli* 5.3 x 10^9/mL)-induced platelet aggregation measured on Multiplate® impedance aggregometry. To the samples were added phosphate-buffered saline (PBS) control, anti-human P-selectin antibody (aP-selectin 20 µg/mL), compstatin (Cp40, 20 µM) or polyclonal sheep control antibody (ctrl.Ab 20 µg/mL), and PBS or *E. coli* as activators. Results are given as the area under the curve (AUC), as mean ± standard deviation (n = 6). #; $p < 0.05$ analyzed using a paired Student's t-test between the samples with PBS and *E. coli*, *; $p < 0.05$ analyzed using one-way ANOVA repeated measurements, and Dunnett's multiple comparisons test, comparing *E. coli*-activated samples with PBS and samples with inhibition or control.

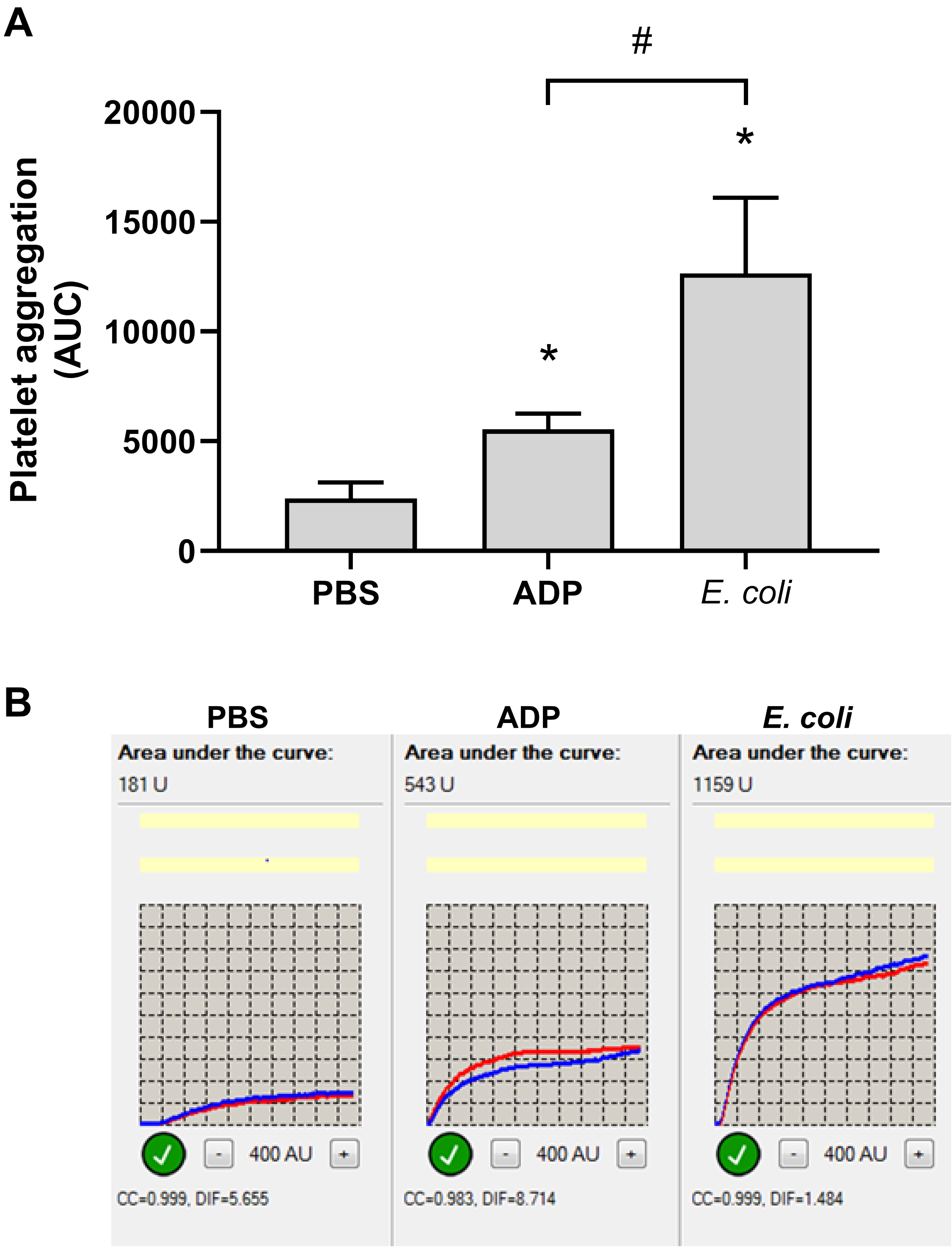

Supplementary Figure 6. The effect of adenosine diphosphate (ADP, 2.1 µM) and *Escherichia coli* (*E. coli*, 5.3 x 10^9/mL) on platelet aggregation in whole blood were compared using Multiplate® impedance aggregometry (**A**). Results are given as the area under the curve (AUC), using mean ± standard deviation (n = 6) #; $p < 0.05$ analyzed using a paired Student's t-test between the samples with ADP and *E. coli*. *; $p < 0.05$ analyzed using one-way ANOVA repeated measurements, and Dunnett's multiple comparisons test, comparing the PBS control to the other samples. The plots show time to platelet aggregation in whole blood for one of the six donors added phosphate-buffered saline (PBS), ADP or *E. coli* (**B**). The aggregation time was set to 30 minutes and results are given as the area under the curve (AUC).

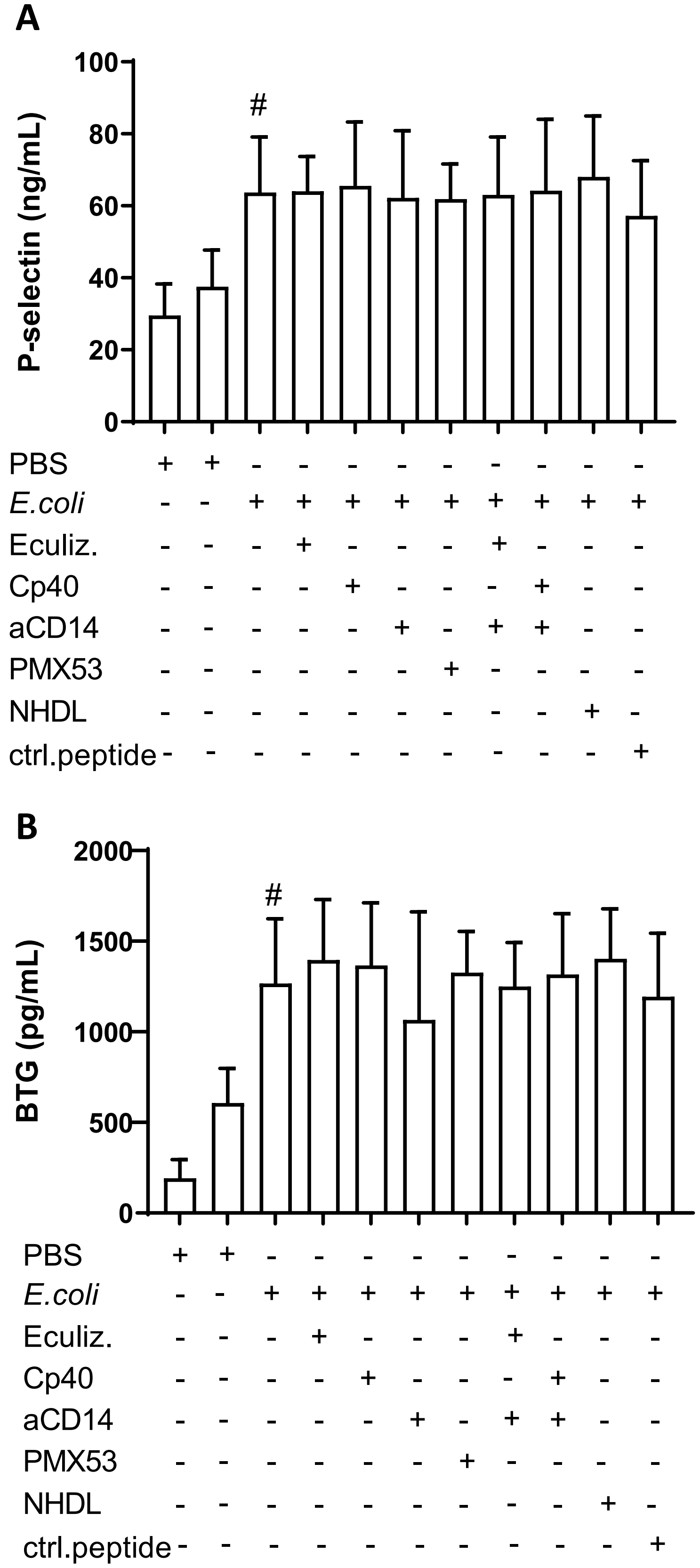

Supplementary Figure 7. The effect of complement inhibitors and anti-CD14 antibody on *Escherichia coli* (*E. coli* 3 x 10^9/mL-induced platelet activation measured as P-selectin (A) and β-thromboglobulin (βTG) (B) using ELISA. To the samples were added phosphate-buffered saline (PBS) control, eculizumab (Eculiz, 100 µg/mL), compstatin (Cp40, 20µM), anti-CD14 antibody (aCD14, 15 µg/mL), the C5aR antagonist PMX53 (10 µM), the IgG2/4 control antibody (NHDL, 15 µg/mL) or control peptide (ctrl.peptide 20 µM), and PBS or *E. coli* as activators. The samples were blocked immediately (the first column) or incubated for 30 minutes before blocking by adding EDTA. The samples were centrifuged and plasma frozen until analyzed. Results are given as mean ± standard deviation (**A**) and median ± interquartile range (**B**) (n = 6). #; $p < 0.05$ analyzed using a paired Student's t-test or Wilcoxon test between the samples with PBS and *E. coli*. The P-selectin and βTG results were reported in ng/mL and pg/mL, respectively.
